# Generalized host‐plant feeding can hide sterol‐specialized foraging behaviors in bee–plant interactions

**DOI:** 10.1002/ece3.5868

**Published:** 2019-12-14

**Authors:** Maryse Vanderplanck, Pierre‐Laurent Zerck, Georges Lognay, Denis Michez

**Affiliations:** ^1^ Laboratory of Zoology Research Institute for Biosciences University of Mons Mons Belgium; ^2^ Evo‐Eco‐Paleo ‐ UMR 8198 CNRS University of Lille Lille France; ^3^ Laboratory of Analytical Chemistry Gembloux Agro‐Bio Tech University of Liège Gembloux Belgium

**Keywords:** bee–flower interactions, generalization, insects, physiological constraints, sterols

## Abstract

Host‐plant selection is a key factor driving the ecology and evolution of insects. While the majority of phytophagous insects is highly host specific, generalist behavior is quite widespread among bees and presumably involves physiological adaptations that remain largely unexplored. However, floral visitation patterns suggest that generalist bees do not forage randomly on all available resources. While resource availability and accessibility as well as nectar composition have been widely explored, pollen chemistry could also have an impact on the range of suitable host‐plants. This study focuses on particular pollen nutrients that cannot be synthesized de novo by insects but are key compounds of cell membranes and the precursor for molting process: the sterols. We compared the sterol composition of pollen from the main host‐plants of three generalist bees: *Anthophora plumipes*, *Colletes cunicularius*, and *Osmia cornuta*, as well as one specialist bee *Andrena vaga*. We also analyzed the sterols of their brood cell provisions, the tissues of larvae and nonemerged females to determine which sterols are used by the different species. Our results show that sterols are not used accordingly to foraging strategy: Both the specialist species *A. vaga* and the generalist species *C. cunicularius* might metabolize a rare C_27_ sterol, while the two generalist species *A. plumipes* and *O. cornuta* might rather use a very common C_28_ sterol. Our results suggest that shared sterolic compounds among plant species could facilitate the exploitation of multiple host‐plants by *A. plumipes* and *O. cornuta* whereas the generalist *C. cunicularius* might be more constrained due to its physiological requirements of a more uncommon dietary sterol. Our findings suggest that a bee displaying a generalist foraging behavior may sometimes hide a sterol‐specialized species. This evidence challenges the hypothesis that all generalist free‐living bee species are all able to develop on a wide range of different pollen types.

## Graphical abstract

We compared the sterol composition of pollen from the main host‐plants of three generalist bees: *Anthophora plumipes*, *Colletes cunicularius*, and *Osmia cornuta*, as well as one specialist bee *Andrena vaga*. We also analyzed the sterols of their brood cell provisions, the tissues of larvae, and nonemerged females to determine which sterols are used by the different species. Our findings suggest that a bee displaying a generalist foraging behavior might sometimes hide a sterol specialized species.

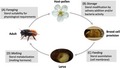

## 1 INTRODUCTION

Plant–insect interactions range from antagonism to mutualism and from specialization to generalization (Mayhew, 1997; Wcislo & Cane, 1996, and references therein; Lengyel, Gove, Latimer, Majer, & Dunn, 2009). While generalist species exploit plants from more than one family, the majority of phytophagous insects is highly host specific relying on a single genus, subfamily or family of plants for their development (Bernays & Chapman, 1994). Specialization among plant‐feeding insects could be partly explained by the limited neural capacity to forage on diverse plant species with different morphologies and by the physiological challenge of digesting tissues from unrelated plants (Janz & Nylin, 2008). Whereas specialization offers obvious evolutionary advantages such as physiological efficiency, optimal foraging, and efficient host discrimination (Janz & Nylin, 2008, and references therein), a minority of herbivorous insects has taken a different evolutionary route and exploits numerous host plants. Such generalist behavior involves processing multiple sensory and chemical signals (Bernays, 2001; Riffell, 2011) and also potentially requires adaptations related to host recognition, foraging, and digestion (Finlay‐Doney & Walter, 2012). However, ecological generalization does not imply that generalists forage randomly on all available plants (Praz, Müller, & Dorn, 2008; Sedivy, Müller, & Dorn, 2011; Thorsteinson, 1960). They can exploit plants to which they might be preadapted behaviorally and/or ecologically (Haider, Dorn, & Müler, 2013; Janz & Nylin, 2008) or forage on multiple hosts that individually fill only a part of their physiological requirements. In the present work, we explore how generalist bees may be partly constrained in their floral choices by their physiological requirements and by the chemical composition of their pollen host.

While <10% of all herbivorous insects feed on plants belonging to more than three different plant families (Bernays & Graham, 1988), half of all free‐living bee species (i.e., excluding cuckoo bees) forage on a wide range of host plants (e.g., *Colletes nigricans* visiting the flowers of up to 15 different plant families; Müller & Kuhlmann, 2008), making bees a pertinent model to understand ecological generalization. Bees rely on floral resources for their development, principally on pollen and nectar (Michener, 2007). Several studies have provided evidence that generalist bees face high interspecific variation in pollen composition (e.g., alkaloids; Gosselin et al., 2013; essential amino acids; Weiner, Hilpert, Werner, Linsenmair, & Bluthgen, 2010; sterols; Vanderplanck et al., 2018) and do not show equivalent development on all pollen diets, with for instance an increase of larval mortality and a decrease in the mass of individual offspring on Asteraceae pollen (e.g., Levin & Haydak, 1957; Sedivy et al., 2011; Vanderplanck et al., 2018). Pollen nutritional content may consequently represent an important constraint in host‐plant selection for generalist bees. While attention has mainly been paid to proteins and amino acids, sterols remain poorly studied despite their importance for numerous physiological processes in bees (e.g., pupation, ovary development) (Behmer & Nes, 2003; Cohen, 2004). Sterols are requisite nutrients since insects cannot synthesize these essential components de nova for hormone production, gene expression, and cell membrane stability (Behmer & Nes, 2003; Cohen, 2004). While cholesterol (C_27_H_46_O) is typically used as the primary sterol, plant phytosterols (C_28_ or C_29_) are not directly used because of their additional carbon(s) (Behmer & Nes, 2003). Physiological pathways of conversion from phytosterols to cholesterol (i.e., dealkylation) occur in basal clades of the Hymenoptera (i.e., Symphyta), but more derived members like bees seem to have lost this ability (Behmer & Nes, 2003). Alternative pathways could be used to cope with this lack of cholesterol such as mutualistic interactions with endosymbiots or synthesis of particular molting hormones (e.g., makisterone A with an additional carbon) from particular phytosterols (Behmer & Nes, 2003; Cohen, 2004).

**Figure 1:**
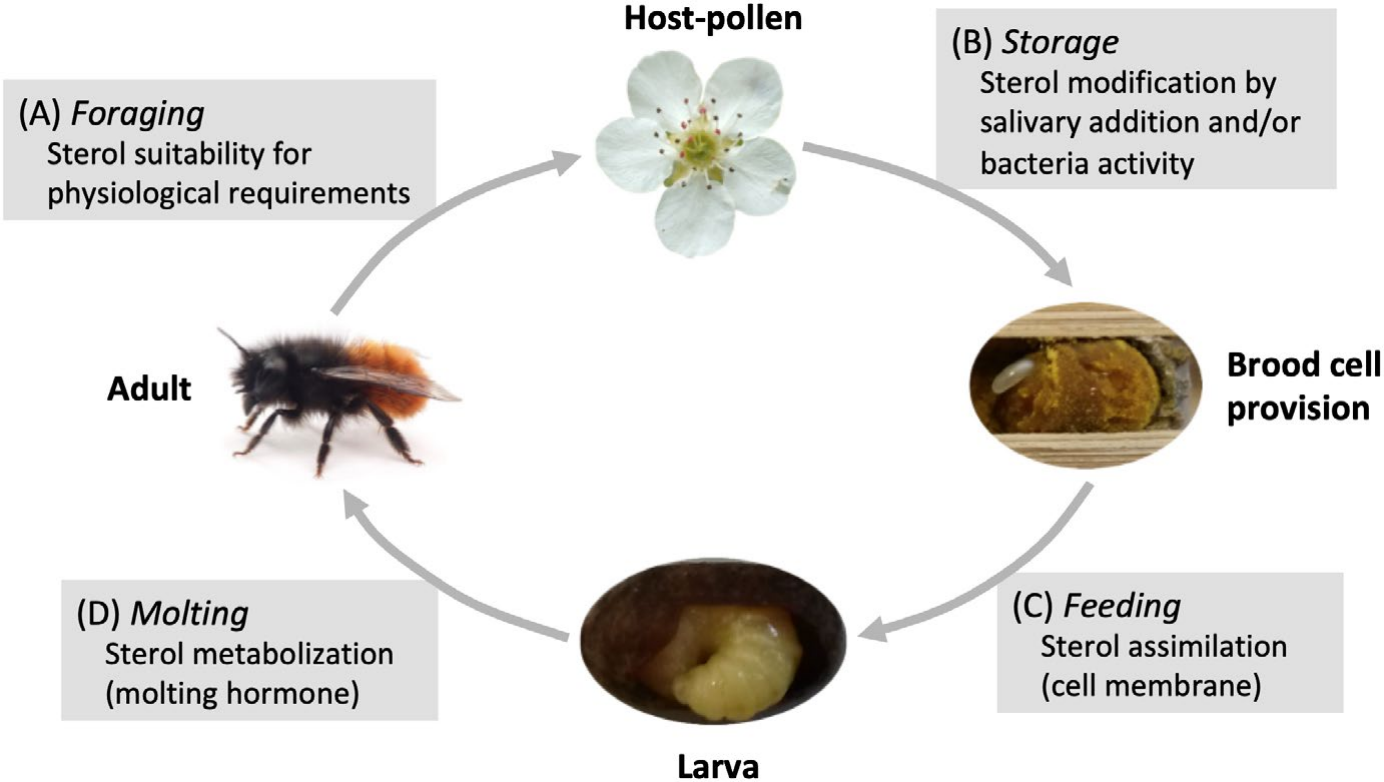
The importance of pollen sterols for host‐plant selection and the life cycle of a bee, here illustrated for the generalist bee *Osmia cornuta*. The main steps of sterol modification, assimilation, and metabolization are indicated in the gray boxes

To evaluate the importance of sterols in bee host‐plant interaction and the sterolic requirement of bees (Figure 1a,b), we compared the sterol profiles of host pollen and brood cell provisions of three generalist bees (*Anthophora plumipes*, Apidae; *Colletes cunicularius*, Colletidae and *Osmia cornuta*, Megachilidae) and one specialist bee (*Andrena vaga*, Andrenidae). Additionally, we aimed to identify (a) the sterols that are assimilated by larvae during pollen feeding by investigating sterol profiles of larval tissues, and (b) the sterols that are metabolized for molting by comparing sterol profiles of tissues of larvae and nonemerged females (Figure 1c,d). We expect generalist bees to forage on host‐pollen that shows a common sterol profile and/or that they are able to metabolize different sterols. On the contrary, the specialist species could forage on host‐pollen that has a peculiar sterol profile and could be constrained to metabolize just one specific sterol.

## 2 MATERIALS AND METHODS

### 2.1 Bee species and plant species

**Figure 2:**
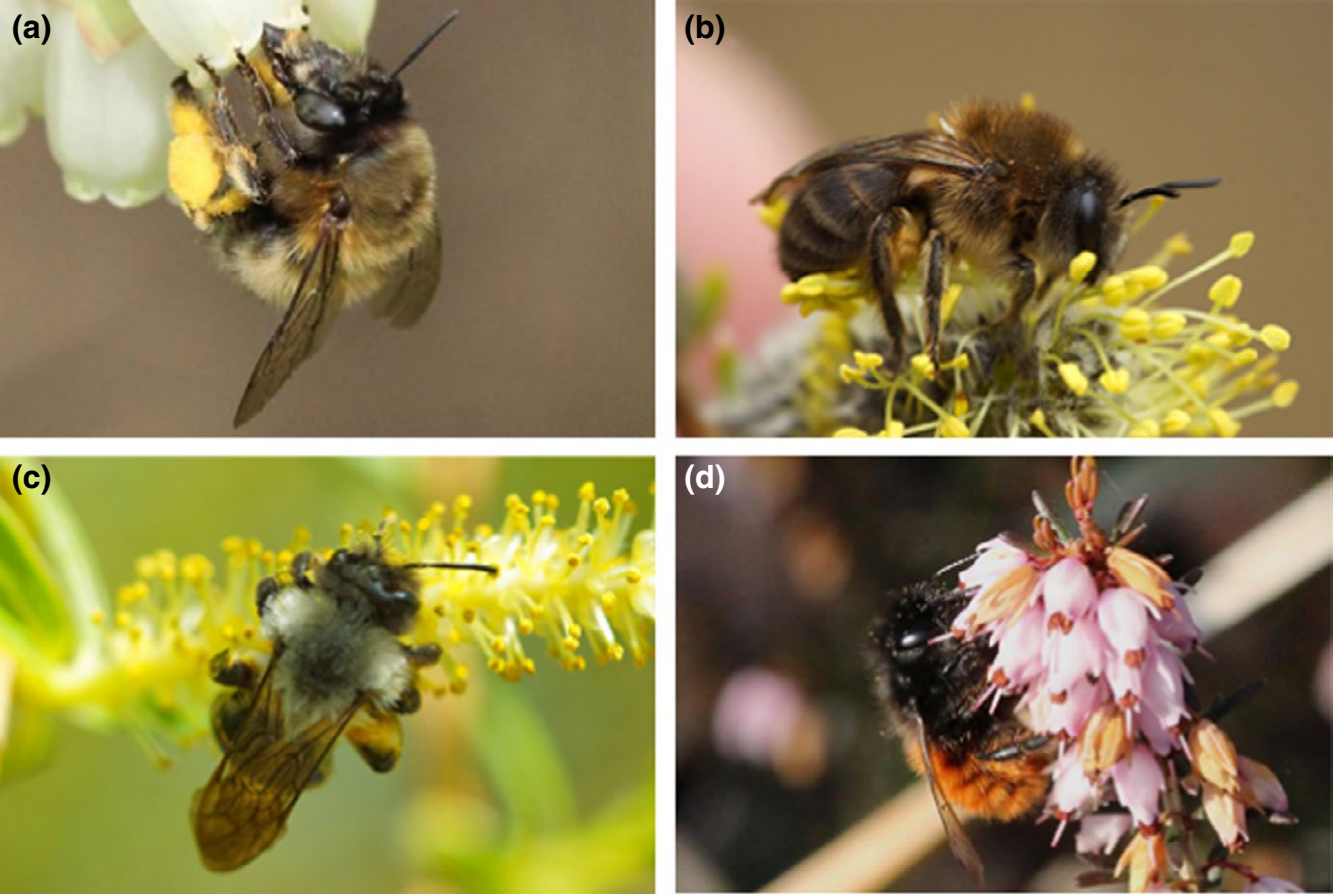
Bees on one of their preferred host‐plants. (a) *Anthophora plumipes* on *Muscari botryoides* (photograph by Kurt Geeraerts), (b) *Colletes cunicularius* on *Salix caprea* (photograph by Henk Wallays), (c) *Andrena vaga* on *Salix fragilis* (photograph by Maxime Drossart), and (d) *Osmia cornuta* on *Erica* sp. (photograph by Kurt Geeraerts)

We selected four spring univoltine bee species that are common in Belgium: (a) *A. vaga* (Andrenidae), a specialist on willow; (b) three generalist species: *A. plumipes* (Apidae), *C. cunicularius* (Colletidae), and *O. cornuta* (Megachilidae) (Figure 2). These solitary species can live in the same habitat and therefore potentially have access to the same plant community. They belong to phylogenetically distant bee lineages (Danforth, Cardinal, Praz, Almeida, & Michez, 2013) and display different life‐history traits.

*Andrena vaga* Panzer (Andrenidae) is a pollen specialist bee that becomes active between mid‐March and the beginning of May (Rezkova, Zakova, Zakova, & Straka, 2012; Westrich, 1989). Females collect large pollen loads predominantly on *Salix* trees (Bischoff, Feltgen, & Breckner, 2003; Vanderplanck, Bruneau, & Michez, 2009; Westrich, 1989). *Andrena vaga* builds deep nests in sandy soils in sunny locations (Westrich, 1989).

*Anthophora plumipes* (Pallas) (Apidae) is a pollen generalist that is active between the end of April and the beginning of June (Westrich, 1989). Females make short and frequent foraging flights and collect small pollen loads from a wide range of characteristically deep‐throated flowers species from Berberidaceae, Boraginaceae (e.g., *Pulmonaria officinalis*, *Symphytum officinale*), Fabaceae (e.g., *Trifolium repens*), Iridaceae, Lamiaceae (e.g., *Lamium album*, *Lamium purpureum*, *Glechoma hederacea*), Liliaceae, Papaveraceae (*Corydalis* sp.), Primulaceae (*Primula* sp.), Rosaceae, and Scophulariaceae (Westrich, 1989). However, it has a strong preference for Lamiaceae. *Anthophora plumipes* builds shallow nests in dry soil with an open entrance protected from the rain.

*Colletes cunicularius* (L.) (Colletidae) is a pollen generalist that is active in early spring from March to May (Bischoff et al., 2003). Females mainly exploit *Salix* spp. (Salicaceae) but also collect pollen from alternative host‐plants, including Asteraceae (Cichorioideae), Brassicaceae, Cistaceae, Ericaceae, Fabaceae (*Cytisus* sp. and *Ilex* sp.), Grossulariaceae, Resedaceae, Rhamnaceae, Rosaceae (*Prunus* sp., *Sorbus* sp. and *Pyrus* sp.), and Sapindaceae (*Acer* sp.) (Müller & Kuhlmann, 2008). This exploitation of alternative host‐plants occurs particularly toward the end of the relatively short flowering season of willows (Bischoff et al., 2003; Müller & Kuhlmann, 2008). *Colletes cunicularius* builds deep nests in sandy soil in sunny locations and is commonly observed in syntopy with *A. vaga* (Müller, Krebs, & Amiet, 1997; Vereecken, Toffin, Gosselin, & Michez, 2006; Westrich, 1989).

*Osmia cornuta* (Latreille) (Megachilidae) is a pollen generalist that has an early seasonal flight period that usually lasts from the beginning of March until the beginning of May (Westrich, 1989). Females collect pollen from plants belonging to many different families including Brassicaceae, Ericaceae, Papaveraceae (*Corydalis* sp.), Ranunculaceae (*Anemone* sp.), Rosaceae (e.g., *Pyrus* sp.), Salicaceae (*Salix* sp.), Sapindaceae (*Acer* sp.), and some monocots (Haider, Dorn, Sedivy, & Müller, 2014; Marquez, Bosch, & Vicens, 1994; Westrich, 1989). *Osmia cornuta *nests in a great variety of preexisting cavities (e.g., in the wall or hollow bamboo stalks) where it brought mud as construction material (Westrich, 1989).

We selected four main host plants for each pollen generalist as well as two widespread and common willow species for the pollen specialist (Table 1). Pollen was collected from the stamens of different flowers by using a turning fork (around 100 mg of fresh pollen) and cleaned under a binocular microscope (i.e., removal of trichomes, anthers, dust, or filaments). All the plants were collected during the bee flying period and from the same area (i.e., one population) to avoid intraspecific variation. During the flying period, we collected brood cell provisions for each species by digging up the nest (for the three ground nesting species: *A. vaga*, *A. plumipes*, and *C. cunicularius*) or by opening bamboo stalks (for the stem nesting species: *O. cornuta*). The brood cell provisions were collected from closed nest cells with an egg on top. We avoided analyzing brood cell provisions with developed larvae. We collected new cells to sample larvae (nondefecating larval stage) 1 month after the flying period, whereas nonemerged females were collected at least 2 months latter (i.e., overwintering diapause as adults). At least three nests per species were used for each sampling session (i.e., brood cell provisions, larvae, nonemerged females) to yield enough material. Moreover, we ensured that several host‐plants were available near the nesting site of generalist species to allow for mixing behavior and avoid biased data (Table 1).

**Table 1:** Four selected bee species, sampled localities, degree of floral specialization, and the main host‐plant from the sampled population. Classification APGIII (2009)

Bee species	Locality	Specialization	Plant species analyzed
*Andrena vaga*	Belgium, Blaton	Specialist (Westrich, 1989)	*Salix caprea* (Salicaceae) *Salix fragilis* (Salicaceae)
*Anthophora plumipes*	Belgium, Malines	Generalist (10 plant families; Westrich, 1989)	*Lamium album* (Lamiaceae) *Pulmonaria officinalis* (Boraginaceae) *Symphytum officinale* (Boraginaceae) *Salix caprea* (Salicaceae)
*Colletes cunicularius*	Belgium, Blaton	Generalist (11 plant families; Müller & Kuhlmann, 2008)	*Cytisus scoparius* (Fabaceae) *Prunus avium* (Rosaceae) *Salix caprea* (Salicaceae) *Sorbus aucuparia* (Rosaceae)
*Osmia cornuta*	Belgium, Mons	Generalist (8 plant families; Westrich, 1989)	*Erica carnea* (Ericaceae) *Muscari botryoïdes* (Asparagaceae) *Pyrus communis* (Rosaceae) *Salix caprea* (Salicaceae)

### 2.2 Sterol analyses

Before each analysis, lyophilized floral pollen and fresh brood cell provisions were carefully homogenized and divided into a minimum of three samples (i.e., 20 mg per analytical replicate). We also removed blind guts from the larvae to avoid bias due to pollen remains as well as wings and legs (mainly chitin) from the female bodies prior to analyses (i.e., single individual per analytical replicate).

Sterols were quantified by GC‐FID after extraction and purification according to the method described by Vanderplanck, Michez, Vancraenenbroeck, and Lognay (2011). The multi‐step procedure can be summarized as follows: (a) saponification with 2 M methanolic potassium hydroxide, (b) extraction of the unsaponifiable portion with diethylether and several water washings, (c) solvent evaporation, (d) fractionation of the unsaponifiable portion by TLC, (e) trimethylsilylation of the sterols (scrapped from the silicagel), and (f) separation by GC. The total sterol content was determined considering all peaks above the limit of quantification; (LOQ = 9.6 ng/1.2 µl injected) whose retention time was between cholesterol and betulin (internal standard). Individual sterols were quantified on the basis of peak areas from analyses. Under the present analytical conditions applied, campesterol and 24‐methylenecholesterol co‐eluted. Therefore, the results are pooled for these two compounds. Compounds were identified according to their retention times in comparison with those of sunflower oil as reference. The identifications were corroborated by GC/MS (Vanderplanck et al., 2011).

### 2.3 Data analyses

To determine whether the host‐plants of each bee species have significantly different sterol composition, data were first square root transformed and standardized using the Wisconsin double standardization (“wisconsin” function, R‐package vegan, Oksanen et al., 2018) prior to the multivariate analysis (i.e., bee species as categorical variable with four levels, all host‐plants were considered equally for a given bee species). The Wisconsin double standardization is a method which first standardizes the data by sterol maximum standardization and afterward by sample total standardization (i.e., normalization to percent abundance). We then performed a perMANOVA using the Bray–Curtis dissimilarities as a measure of ecological distance and 999 permutations (“adonis” function, R‐package vegan). An advantage of this method is that the procedure is less dependent on data distribution than constrained methods. When perMANOVA returned significant *p*‐value (*p* < .05), multiple pairwise comparisons were conducted on the data to detect precisely the differences and *p*‐values were adjusted using Bonferroni's correction to avoid increases of type error I due to multiple testing. Indicator Species Analyses (Indval; Dufrêne & Legendre, 1997) were finally performed using the “indval” function from the labdsv package (Roberts, 2012) to identify the pollen sterols that were indicator of host‐plants exploited by a given bee species. This analysis calculates an indicator value based on relative abundance (specificity) and relative frequency (fidelity) for each sterol to identify the compound(s) with the highest indicator value for each sample. A *p*‐value was calculated for each sterol‐bee combination to assess whether pollen sterols are significantly found in association with a given bee species. All *p*‐values were adjusted using Holm's correction, to avoid increases of type error I due to multiple testing. Both similarities and dissimilarities were visually assessed on a nonmetric multidimensional scaling (nMDS) ordination using the “metaMDS” function from the package vegan. This function transforms the data using the Wisconsin double standardization, applies Bray–Curtis dissimilarities, runs NMDS multiple times with random starts to avoid local optima, and rotates the axes of the final configuration so that the variance of points is maximized on the first dimension. We determined the appropriate number of axes to use by obtaining stress values for ten replicates NMDS runs for each number of dimensions between one and four. We set the maximum number of random starts for each run at 500. For the final number of dimensions, we selected the lowest number of axes that had a stress value ≤0.2 (conventional cutoff; McCune & Grace, 2002). Similar statistical procedure was used to compare the host‐pollen (i.e., plant species as categorical variable with 11 levels), brood cell provisions, tissues of larvae, and nonemerged females among the four bee species (i.e., bee species as categorical variable with four levels).

To detect sterol(s) that might be involved in growth and developmental processes of the selected bee species, we compared the tissues from larvae and nonemerged females for each bee species using a perMANOVA on transformed data (i.e., square root and Wisconsin double standardization). Similarity percentage analyses were then performed in R using the “simper” function from the vegan package to identify the compounds that were responsible for detected differences between larvae and nonemerged females. Statistical results were summarized and displayed on back‐to‐back horizontal bar plots. All data analyses and visualization were performed in R version 3.4.0 (R Core Team, 2017).

## 3 RESULTS

### 3.1 Sterols in pollen and brood cell provisions

Pollen of the 11 targeted floral species displayed concentrations of total sterols ranging from 1.17 (*Muscari botryoides*) to 26.85 (*Pyrus communis*) mg per g of lyophilized matter (*F*_10,39_ = 23.13, *p* < .001, Table 2). Pollen from *Pyrus communis*, *Prunus avium*, and *Pulmonaria officinalis* showed a significantly higher sterol concentration than pollen from *Muscari botryoides*, *Erica carnea*, and *Lamium album* whereas the other species displayed intermediate concentrations (Table 2). Pollen of Ericaceae (*E. carnea*), Fabaceae (*Cytisus scoparius*), Lamiaceae (*L. album*), and Salicaceae (*Salix caprea* and *Salix fragilis*) displayed high concentrations of C_29_ sterols (β‐sitosterol and δ5‐avenasterol) whereas 24‐methylenecholesterol (C_28_ sterol) and campesterol (same fraction) are the most abundant sterolic consituents in Boraginaceae (*P. officinalis* and *Symphytum officinale*), Asparagaceae (*M. botryoides*), and Rosaceae (*P. avium*, *P. communis* and *Sorbus aucuparia*), followed by δ5‐avenasterol and β‐sitosterol (Table 2). The analyses show that all plants significantly differed from each other (*F*_10,39_ = 21.15, *p* < .001; multiple pairwise comparisons, *p* < .05), except *P. avium*, *S. aucuparia*, and *S. officinale* whose pollen displayed similar phytosterolic composition (multiple pairwise comparisons, *p* > .05). The occurrence of δ7‐avenasterol in pollen is indicative of *E. carnea* (*p* = .009, indicator value = 0.254) while δ5‐avenasterol in pollen is indicative of *L. album* (*p* = .009, indicator value = 0.186), 24‐methylenecholesterol and stigmasterol of *P. officinalis* (24‐methylenecholesterol: *p* = .009, indicator value = 0.269; stigmasterol: *p* = .015, indicator value = 0.269), and cholestenone of *S. fragilis* (*p* = .009, indicator value = 0.315).

**Table 2:** Sterolic compositions of floral pollen from the 11 plant models

Species	*n*	Cholesterol	Desmosterol	24‐methylenechol. and campesterol	Stigmasterol	β‐sitosterol	δ5‐avenasterol	Cholestenone	δ7‐stigmasterol	δ7‐avenasterol	Total content (mg/g)
*Cytisus scoparius*	5	2.48 ± 0.32	0.09 ± 0.09	**17.33 ± 3.27**	0.80 ± 0.35	**46.76 ± 4.03**	**30.13 ± 1.36**	1.10 ± 1.27	0.75 ± 1.03	0.56 ± 0.11	5.24 ± 0.72
*Erica carnea*	5	3.16 ± 0.55	1.16 ± 0.92	**7.63 ± 0.66**	0.97 ± 0.59	**54.09 ± 5.33**	**19.52 ± 1.40**	0.21 ± 0.29	**7.03 ± 2.11**	**6.23 ± 1.40**	1.79 ± 0.59
*Lamium album*	5	**6.10 ± 0.90**	1.73 ± 1.66	4.77 ± 2.31	0.05 ± 0.11	**44.66 ± 2.95**	**41.87 ± 2.66**	0.65 ± 0.61	0.00 ± 0.00	0.18 ± 0.24	3.06 ± 1.41
*Muscari botryoides*	5	**10.37 ± 7.05**	2.32 ± 0.90	**40.74 ± 8.08**	4.27 ± 1.27	**28.09 ± 7.19**	**5.40 ± 1.72**	0.00 ± 0.00	**7.19 ± 2.61**	1.64 ± 0.51	1.17 ± 0.39
*Prunus avium*	3	0.37 ± 0.07	0.03 ± 0.03	**89.92 ± 1.00**	1.13 ± 0.11	**5.74 ± 0.66**	2.47 ± 0.13	0.23 ± 0.10	0.00 ± 0.00	0.11 ± 0.05	11.94 ± 0.47
*Pulmonaria officinalis*	5	**2.95 ± 0.15**	0.36 ± 0.03	**88.97 ± 1.14**	2.14 ± 1.07	**2.94 ± 0.20**	2.60 ± 0.16	0.04 ± 0.08	0.00 ± 0.00	0.00 ± 0.00	9.43 ± 1.17
*Pyrus communis*	6	**2.79 ± 2.26**	2.58 ± 2.80	**85.57 ± 3.33**	0.28 ± 0.38	**5.17 ± 1.61**	1.74 ± 1.08	0.45 ± 0.24	1.43 ± 3.06	0.01 ± 0.01	26.85 ± 19.85
*Salix caprea*	5	1.07 ± 0.34	0.22 ± 0.36	**4.89 ± 0.18**	0.33 ± 0.09	**44.41 ± 1.97**	**42.10 ± 1.96**	1.84 ± 0.74	3.22 ± 2.10	1.90 ± 0.42	6.52 ± 0.44
*Salix fragilis*	5	1.07 ± 0.44	0.03 ± 0.02	4.05 ± 0.28	0.09 ± 0.14	**50.08 ± 3.24**	**33.64 ± 2.86**	**5.92 ± 3.71**	2.23 ± 1.51	2.89 ± 1.30	5.59 ± 0.75
*Sorbus aucuparia*	3	2.10 ± 0.42	0.08 ± 0.07	**81.68 ± 4.77**	4.81 ± 8.00	**5.16 ± 0.76**	**4.98 ± 3.94**	0.00 ± 0.00	0.71 ± 0.32	0.48 ± 0.42	8.61 ± 1.83
*Symphytum officinale*	3	1.39 ± 1.11	0.00 ± 0.00	**47.07 ± 15.03**	3.62 ± 1.39	**24.83 ± 9.81**	**16.29 ± 9.01**	0.00 ± 0.00	4.42 ± 3.38	2.39 ± 0.91	9.62 ± 9.81

Note

The concentrations of individual sterols are expressed as percentage of total sterolic content (mean ± *SD*). Major sterols (>5%) are indicated in bold.

**Figure 3:**
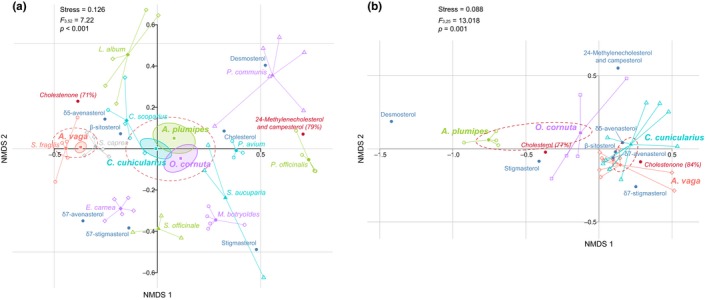
nMDS ordination plot based on Bray–Curtis distances calculated on abundances (mg/g) of sterolic compounds in (a) pollen from the host‐plants, with centroids per plant species and per bee species (*n* are mentioned in Table 2) (b) brood cell provisions, with centroids per bee species (*n* are mentioned in Table 3). Sterols in red are indicative (indicative value in %)

Statistical analysis using the foraging bee species as explicative variable detected a significant difference in pollen phytosterolic composition among the four species (*F*_3,52_ = 7.22, *p* < .001). Pairwise comparisons reveal that the specialist species *A. vaga* significantly differed from the three generalist ones (i.e., *A. plumipes*, *C. cunicularius*, *O. cornuta*). When structuring the data according to the foraging behavior (i.e., generalist species vs. specialist species), a significant association was detected between 24‐methylenecholesterol (C_28_ sterol) and pollen used by generalist bee species (Indicator Compound Analysis, *p* = .009, indicator value = 0.744) whereas occurrence of cholestenone (C_27_ sterol) in pollen is indicative of host plants foraged on by *A. vaga*, the specialist bee species (Indicator Compound Analysis, *p* = .009, indicator value = 0.719; Figure 3a).

The results were slightly different for brood cell provisions (i.e., larval food) with the statistical analyses supporting two groups (*F*_3,25_ = 13.02, *p* = .001; Figure 3b). The first group consists of brood cell provisions of *A. vaga* and *C. cunicularius* that are significantly associated with cholestenone (C_27_ sterol; *p* = .009, indicator value = 0.839) whereas the second group is composed of brood cell provisions of *A. plumipes* and *O. cornuta* that are significantly associated with cholesterol (C_27_ sterol; *p* = .009, indicator value = 0.767; Figure 3b, Table 3).

**Table 3:** Sterolic compositions of brood cell provisions, larval tissues, and nonemerged females

Species	*n*	Cholesterol	Desmosterol	24‐methylenechol. and campesterol	Stigmasterol	β‐sitosterol	δ5‐avenasterol	Cholestenone	δ7‐stigmasterol	δ7‐avenasterol	Total content (mg/g)
*Andrena vaga*											
Brood cell provisions	10	0.59 ± 0.35	0.00 ± 0.00	**3.94 ± 0.41**	0.19 ± 0.13	**61.06 ± 4.14**	**28.17 ± 4.02**	2.40 ± 1.44	2.18 ± 2.99	1.47 ± 1.35	6.046 ± 2.89
Larvae	6	1.48 ± 0.56	0.09 ± 0.07	**12.47 ± 1.96**	0.83 ± 0.32	**42.34 ± 4.63**	**22.26 ± 11.71**	**19.58 ± 9.25**	0.26 ± 0.39	0.68 ± 0.81	0.51 ± 020
Nonemerged females	15	6.07 ± 8.65	0.35 ± 0.22	**15.86 ± 3.78**	0.24 ± 0.83	**32.00 ± 6.37**	**40.06 ± 10.36**	3.48 ± 2.79	0.30 ± 0.97	1.64 ± 3.62	0.84 ± 0.38
*Anthophora plumipes*											
Brood cell provisions	4	3.36 ± 1.52	**31.90 ± 10.43**	**25.43 ± 9.05**	0.79 ± 0.24	**25.20 ± 9.82**	**12.76 ± 5.04**	0.07 ± 0.10	0.32 ± 0.17	0.17 ± 0.12	2.20 ± 0.79
Larvae	5	1.60 ± 0.76	**10.20 ± 12.75**	**55.74 ± 15.44**	0.14 ± 0.20	**18.62 ± 5.65**	**13.45 ± 2.99**	0.14 ± 0.15	0.07 ± 0.10	0.05 ± 0.07	6.30 ± 1.86
Nonemerged females	5	0.77 ± 0.66	0.00 ± 0.00	**73.96 ± 4.77**	**9.25 ± 20.67**	**7.39 ± 2.62**	**8.46 ± 2.84**	0.17 ± 0.38	0.00 ± 0.00	0.00 ± 0.00	1.94 ± 0.77
*Colletes cunicularius*											
Brood cell provisions	10	0.13 ± 0.17	0.01 ± 0.01	**27.07 ± 27.41**	0.14 ± 0.15	**32.88 ± 15.85**	**34.41 ± 11.03**	2.36 ± 1.07	1.01 ± 1.34	2.00 ± 1.85	3.08 ± 0.70
Larvae	6	2.59 ± 1.67	0.07 ± 0.11	**6.51 ± 2.07**	1.05 ± 0.68	**51.99 ± 3.90**	**22.66 ± 10.32**	**9.90 ± 8.82**	4.13 ± 2.75	1.08 ± 1.33	0.81 ± 0.51
Nonemerged females	10	0.91 ± 0.40	0.10 ± 0.11	**8.54 ± 0.46**	0.24 ± 0.12	**63.02 ± 4.16**	**26.07 ± 3.62**	0.74 ± 0.28	0.20 ± 0.26	0.18 ± 0.10	1.22 ± 0.33
*Osmia cornuta*											
Brood cell provisions	5	**7.82 ± 13.02**	0.08 ± 0.19	**40.46 ± 34.86**	0.40 ± 0.33	**25.61 ± 14.37**	**22.60 ± 13.07**	0.41 ± 0.41	0.46 ± 0.30	2.15 ± 1.69	8.77 ± 6.35
Larvae	5	0.91 ± 0.29	0.12 ± 0.15	**51.37 ± 17.36**	0.73 ± 1.31	**32.15 ± 10.11**	**14.00 ± 7.33**	0.29 ± 0.31	0.14 ± 0.09	0.29 ± 0.15	2.98 ± 0.87
Nonemerged females	5	1.64 ± 1.64	0.24 ± 0.11	**31.34 ± 15.68**	0.39 ± 0.18	**31.23 ± 17.92**	**34.56 ± 11.69**	0.10 ± 0.08	0.22 ± 0.14	0.27 ± 0.05	1.02 ± 0.32

Note

The concentrations of individual sterols are expressed as percentage of total sterolic content (mean ± *SD*). Major sterols (>5%) are indicated in bold.

### 3.2 Sterol in bees

#### 3.2.1 Sterol assimilation

Larvae of the four bee species displayed high concentrations of C_29_ sterolic compounds like β‐sitosterol and δ5‐avenasterol in their tissues, as well as high concentrations of 24‐methylenecholesterol (C_28_ sterol) and campesterol (same fraction) compared to the other detected sterols (Table 3). These compounds were also abundant in the brood cell provisions (Table 3) and quite common in pollen (Table 2). Such occurrence in larval and adult tissues from the four bee species suggests their assimilation regardless of the bee specialization (Table 3). Besides these common sterols, tissues of *A. plumipes* larvae also contained high concentration of desmosterol whereas larval tissues of *A. vaga* and *C. cunicularius* contained high concentrations of cholestenone (Table 3).

**Figure 4:**
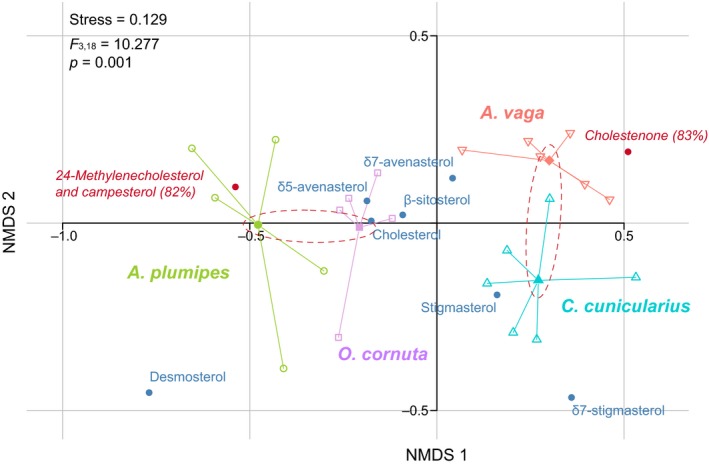
nMDS ordination plot based on Bray–Curtis distances calculated on abundances (mg/g) of sterolic compounds in larval tissues, with centroids per bee species (*n* are mentioned in Table 3). Sterols in red are indicative (indicative value in %)

Statistical analysis detected a significant difference in sterolic composition of larval tissues among the four species (*F*_3,18_ = 10.28, *p* < .001) and the pairwise comparisons structure the data in two distinct groups. The first group consists of the larvae of *A. vaga* and *C. cunicularius* that are significantly associated with cholestenone (C_27_ sterol) (*p* = .009, indicator value = 0.833) whereas the second group is composed of the larvae of *A. plumipes* and *O. cornuta* that are significantly associated with 24‐methylenecholesterol (C_28_ sterol; *p* = .009, indicator value = 0.818; Figure 4, Table 3).

#### 3.2.2 Sterol metabolization

The 24‐methylenecholesterol was the most abundant sterol in both larval tissues and nonemerged females of *A. plumipes*, followed by either stigmasterol (nonemerged females) or β‐sitosterol (larvae), and δ5‐avenasterol. Regarding *O. cornuta*, tissues from both larvae and nonemerged females contained the same major phytosterols, namely 24‐methylenecholesterol, β‐sitosterol, and δ5‐avenasterol, but in slightly different ratios (Table 3). For both *A. vaga* and *C. cunicularius*, the most abundant sterols identified in larval tissues and nonemerged females were β‐sitosterol and δ5‐avenasterol followed by 24‐methylenecholesterol for nonemerged females and cholestenone for larvae (Table 3).

**Figure 5:**
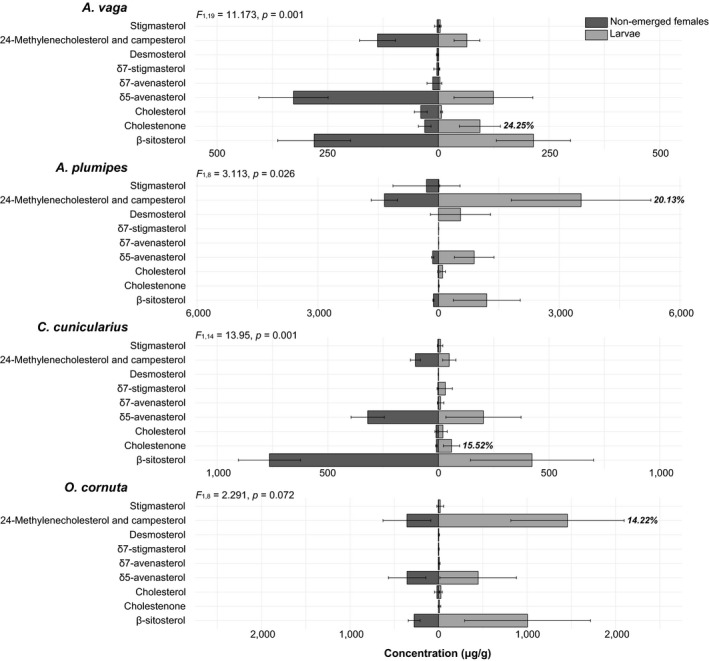
Sterolic compositions (μg/g; mean ± *SD*) of tissues for nonemerged female (dark gray) and larvae (light gray) of *Andrena vaga*, *Anthophora plumipes*, *Colletes cunicularius*, and *Osmia cornuta*. Molecules with the highest percentage contribution (SIMPER) to the defined difference between development stages are given for each bee species (%)

Except for *O. cornuta*, a significant difference was detected between nonemerged females and larvae (*p* < .05). For *A. plumipes*, larval tissues were more concentrated in 24‐methylenecholesterol (SIMPER, contribution to overall dissimilarity: 20.13%). This suggests a metabolization of this C_28_ sterol during the molting (Figure 5). The desmosterol was also more abundant in larvae compared to the nonemerged females but was too variable to be involved in key metabolic pathway such as molting. Despite no significant difference between nonemerged females and larvae was detected for *O. cornuta*, likely because of the high variation in sterol concentration within sample type, the 24‐methylenecholesterol remains an excellent candidate for precursor of molting hormone (SIMPER, contribution to overall dissimilarity: 14.22%) (Figure 5). For both *A. vaga* and *C. cunicularius*, larval tissues were more concentrated in cholestenone (SIMPER, contribution to overall dissimilarity: 24.25% for *A. vaga*; 15.52% for *C. cunicularius*). This suggests a metabolization of this C_27_ during the molting (Figure 5, Table 3). Other sterols were more abundant in larvae compared to the nonemerged females (i.e., stigmasterol for *A. vaga* and δ7‐stigmasterol for *C. cunicularius*) but were too variable to be involved in key metabolic pathway such as molting.

## 4 DISCUSSION

The results show that the sterolic profile of pollen was quite variable among the host‐plant species. However, some sterols were common (β‐sitosterol, δ5‐avenasterol, and 24‐methylenecholesterol) and were also abundant in brood cell provisions as well as in the tissues of larvae and nonemerged females. This highlighted their importance in bee nutrition as well as their assimilation in bee tissues. As bee tissues displayed a sterolic composition similar to the food (i.e., pollen and brood cell provisions), bees are likely to lack dealkylation pathways.

Regarding sterol metabolization, *A. plumipes* and *O. cornuta* might rely on the 24‐methylenecholesterol for the synthesis of their molting hormone. As this pollen sterol is quite spread among plant species, such metabolic pathway would allow them to exploit a large range of host‐plant, so that they could be considered as true generalist species. This is not the case of *C. cunicularius* that seemed to display a similar metabolic pathway than *A. vaga*, the specialist bee species. Both species might likely use the cholestenone, a quite uncommon pollen sterol, as precursor of their molting hormone. Although this sterol was only found in high relative abundance in pollen of *S. fragilis*, these two bee species could be capable of concentrating into their tissues this minor dietary sterol in preference to others that are available in much larger amounts, as it has been already shown for other insect species (reviewed in Clayton, 1964). Despite its apparent generalist foraging behavior, *C. cunicularius* might then be constrained by the dietary sterol it is specialized on. This bee species, while clearly a generalist under the traditional definition, could be considered to have a much more constrained diet than the other two generalists investigated here. All these highlights are hereafter discussed in regards of the extant literature.

### 4.1 The significance of sterol variability in pollen of flowering plants

Like in many plants, the pollen of *M. botryoides*, *P. avium*, *P. officinalis*, *P. communis*, *S. aucuparia*, and *S. officinale* shows a high level of 24‐methylenecholesterol (Lusby, Buchmann, & Feldlaufer, 1993; Nes & Schmidt, 1988; Roger et al., 2016). Occasionally, however, β‐sitosterol is the major sterol (Standifer, Devys, & Barbier, 1968) as we found in *C. scoparius*, *E. carnea*, *L. album*, *S. caprea*, and *S. fragilis*. Despite these differences in major phytosterols, the 11 plant species investigated herein contain the same sterols but in different ratios. Such similarity in pollen composition could allow bees to display a generalist foraging behavior (i.e., foraging on plants from unrelated clades) and might be selected at wide geographic scale since it directly benefits the generalist bees and promotes generalization in pollination systems. However, some plants display particular pollen sterolic profile with large absolute amounts of less conventional sterols such as cholestenone in the pollen of *S. fragilis*. This has been already shown for the pollen of heather (*Calluna vulgaris*, Ericaceae) that contains large amount of stigmasterol, as well as for the pollen of cottonwood (*Populus fremontii*, Salicaceae) and cat's ear (*Hypochoeris radicata*, Asteraceae) (Standifer et al., 1968) that both display high level of cholesterol.

Although *S. caprea* and *S. fragilis* display similar sterolic composition as also observed for species belonging to Rosaceae and, to a lesser extent, to Boraginaceae; Standifer et al. (1968) found little evidence that taxonomy could be used to predict pollen sterol profile since three species belonging to Salicaceae (e.g., *Populus* genus, *Salix* genus) varied widely in the content of C_27_, C_28,_ and C_29_ sterols. This sterolic diversity is not limited to pollen but concerns all plant parts. Little is known about the functional significance of this variation. One hypothesis is that sterol profiles may reflect adaptations to local abiotic conditions but this explanation is not always sufficent (Behmer & Nes, 2003). Another hypothesis is that phytosterol profiles may function as a unique defence against insect herbivores, for example, in grasshoppers (Behmer & Nes, 2003). Sterolic composition of pollen, and more globally pollen nutrients, may therefore affect both generalist and specialist bee species (Gosselin et al., 2013; Praz et al., 2008; Sedivy et al., 2011; Weiner et al., 2010).

One strong hypothesis is that particular sterolic compounds could filter through the available spectrum of floral visitors (i.e., nutritional compound for effective pollinators and toxic repellent for robbers or noneffective visitors) and thereby promote tight association with obligate specialists. Such specialization in pollination systems presents advantages for both bees and plants since it reduces pollinator competition and improves plant pollination efficiency by restricting the range of visitors to a specialist guild (Suzuki, Dohzono, & Hiei, 2007). Other floral traits are known to support pollination specificity such as nectar and floral scent (Johnson, Hargreaves, & Brown, 2006; Shuttleworth & Johnson, 2009). Growing evidence suggests that pollination syndromes are not limited to morphological traits but convergent suites of floral chemical traits could also act as filters in host‐plant selection and therefore pollination systems (Johnson et al., 2006; Shuttleworth & Johnson, 2009; Vanderplanck et al., 2017; Weiner et al., 2010). This hypothesis is strongly supported by the bee abilities to detect pollen nutritional quality and discriminate among hosts (Vaudo, Patch, Mortensen, Tooker, & Grozinger, 2016). Several studies have shown that bumblebees preferentially forage on plant species providing protein‐rich pollen (Hanley, Franco, Pichon, Darvill, & Goulson, 2008; Kitaoka & Nieh, 2009; Leonhardt & Blüthgen, 2012; Rasheed & Harder, 1997; Robertson, Mountjoy, Faulkner, Roberts, & Macnair, 1999). The composition and concentration in amino acids also seem to impact foraging decision and behavior of bees (Alm, Ohnmeiss, Lanza, & Vriesenga, 1990; Hanley et al., 2008; Leonhardt & Blüthgen, 2012; Weiner et al., 2010). Moreover, addition of lipid extracts from pollen to substitutes such as cellulose powder has been shown to stimulate pollen foraging in honeybee (Pernal & Currie, 2002). Such active extracts are known to contain phytosterols or steroids (Hügel, 1962; Louveaux, 1959) as well as free fatty acids (Hopkins, Jevans, & Boch, 1969; Lepage & Boch, 1968), strengthening the hypothesis of a potential role of pollen sterol in bee foraging decision.

### 4.2 The pollen sterols as a constrain for floral choices

High concentrations of β‐sitosterol, δ5‐avenasterol, and 24‐methylenecholesterol were found in larval and adult tissues of the four bee species, suggesting their assimilation and involvement in structural roles such as membrane inserts (Behmer & Nes, 2003). Because these phytosterols are common in pollen of a wide taxonomic range of angiosperms (Barbier, Hügel, & Lederer, 1960; Lusby et al., 1993; Nes & Schmidt, 1988; Standifer et al., 1968), they might not represent a constraint for host‐plant selection. As the sterolic composition of larval and adult tissues reflects this one of the food resources (i.e., floral pollen and brood cell provisions), the four bee species do probably not dealkylate their dietary sterols.

The comparison between larvae and nonemerged females suggested that cholestenone (C_27_ sterol) would be a good candidate for precursor of molting hormone in *A. vaga* and *C. cunicularius* whereas 24‐methylenecholesterol would be used as precursor of molting hormone in *A. plumipes* and *O. cornuta*. Regarding these results, we may suggest that *A. vaga* and *C. cunicularius* could synthesize a C_27_ ecdysteroid such as 20‐hydroxyecdysone using cholestenone as a precursor while *A. plumipes* and *O. cornuta* could rather synthesize a C_28_ ecdysteroid such as makisterone A. Such implication of the cholestenone (C_27_ sterol) in the 20‐hydroxyecdysone synthesis has been already described in *Manduca sexta* (Grieneisen, Warren, & Gilbert, 1993, and references therein) whereas the use of alternative phytosterols (i.e., C_28_ sterols) to synthesize makisterone A for molting has been described for *Drosophila melanogaster* (Redfern, 1986) and *Bombus terrestris* (Regali, 1996). Although our data are very suggestive, experiments involving radioactive labeling techniques and dietary supply remain nevertheless needed to fully validate our interpretation and demonstrate the dietary requirement for a given sterol in the four bee species.

In light of previous similar studies, bees seem to have a sterolic physiology that is relatively conserved since all bee models studied to date lack dealkylation ability and use C_27_ or C_28_ as precursor of 20‐hydroxyecdysone or makisterone A, respectively (*Apis mellifera*, *Megachile rotundata*, *Diadasia rinconis* and *B. terrestris*; in Feldlaufer, Herbert, Svoboda, & Thompson, 1986; Svoboda & Lusby, 1986; Feldlaufer, Lusby, Weirich, Svoboda, & Buchmann, 1993 and Regali, 1996, respectively). This could be partly explained by their ecological specialization on pollen. Other insect groups such as flies display higher diversity in their ecologies and sterol physiologies. For example, the fruit fly *D. melanogaster* is an ecological generalist but other *Drosophila* species are specialized on fruit, mushrooms, cacti, flowers, or even the excretory pores of land crabs (Markow & O'Grady, 2005). Among these specialist flies, *Drosophila pachea* represents an unprecedented model since it is specialized on senita cacti (*Lophocereus schottii*, Cactaceae) and requires an uncommon dietary sterol from its host plant, the lathosterol (Kircher, Heed, Russell, & Groove, 1967). Lang et al. (2012) showed that *D. pachea* evolved an obligate specialization on senita cacti through changes in a single enzyme. Even for the generalist grasshopper *Schistocerca americana*, metabolic constraints presumably restrict the spectrum of phytosterols capable of supporting normal growth and development (Behmer & Elias, 1999). These sterol metabolic constraints are a shared trait among grasshopper species that suffer high levels of mortality when they accumulate unsuitable sterols (Behmer & Elias, 1999, 2000), which may also occur in bees (Vanderplanck et al., 2018).

Overall our results show that shared sterolic profiles among floral species could facilitate exploitation of a wide range of host‐plants by two generalist bees (*A. plumipes* and *O. cornuta*) but that the generalist *C. cunicularius* might be more constrained in its floral choices by a quite rare dietary sterol. In this regard, *C. cunicularius* might share a similar sterol requirement with the specialist *A. vaga* and not with the other generalist bees, which could be verify using dietary supply experiments as well as isotopic tracer techniques (reviewed in Clayton, 1964). Our findings suggest that bees with a generalist foraging pattern such as *C. cunicularius* could hide sterol specialists that might be highly specific in terms of sterol preferences, irrespective of the plant taxonomy, assuming the hypothesis of the improbability of ecological generalization in nature (Loxdale, Lushai, & Harvey, 2011).

## CONFLICT OF INTEREST

None declared.

## AUTHORS' CONTRIBUTIONS

DM conceived and designed the study. MV coordinated the study and carried out laboratory work with the help of P‐LZ. MV, P‐LZ, and GL participated in data analysis. MV carried out the statistical analyses and drafted the manuscript. DM and GL critically revised the manuscript. All authors gave final approval for publication and agree to be held accountable for the work performed therein.

## Data Availability

Data supporting the results: Dryad: https://doi.org/10.5061/dryad.cz8w9gj03.
